# Stearoyl-CoA desaturase-1 mediated cell apoptosis in colorectal cancer by promoting ceramide synthesis

**DOI:** 10.1038/srep19665

**Published:** 2016-01-27

**Authors:** Ling Chen, Jie Ren, Longhe Yang, Yanting Li, Jin Fu, Yuhang Li, Yifeng Tian, Funan Qiu, Zuguo Liu, Yan Qiu

**Affiliations:** 1 Medical College, Xiamen University, Xiamen, Fujian 361000, P. R. China; 2 Clinical Research Institute, The First Affiliated Hospital, University of South China, Hengyang, Hunan 421000, P. R. China; 3 Engineering Research Center of Marine Biological Resource Comprehensive Utilization, Third Institute of Oceanography, State Oceanic Administration, Xiamen, Fujian 361000, P. R. China; 4 Department of Hepatobiliary Surgery, Fujian Provincial Hospital, Fuzhou, Fujian 350001, P. R. China

## Abstract

Inhibition of stearoyl-CoA desaturase 1 (SCD1) has been found to effectively suppress tumor cell proliferation and induce apoptosis in numerous neoplastic lesions. However, mechanism underlying SCD1-mediated anti-tumor effect has maintained unclear. Herein, we reported endo-lipid messenger ceramides played a critical role in tumor fate modulated by SCD1 inhibition. *In vitro* study in colorectal cancer cells demonstrated inhibition of SCD1 activity promoted apoptosis attributed to mitochondria dysfunctions, upregulation of reaction oxygen species (ROS), alteration of mitochondrial transmembrane potential and translocation of mitochondrial protein cytochrome C. While these effects were mediated by intracellular ceramide signals through induction of ceramide biosynthesis, rather than exclusive SFA accumulation. *In vivo* study in xenograft colorectal cancer mice showed pharmacologic administration of SCD1 inhibitor A939 significantly delayed tumor growth, which was reversed by L-cycloserine, an inhibitor of ceramide biosynthesis. These results depicted the cross-talk of SCD1-mediated lipid pathway and endo-ceramide biosynthesis pathway, indicating roles of ceramide signals in SCD1-mediated anti-tumor property.

Constitutive activation of the fatty acid biosynthetic pathway, which produces saturated fatty acids (SFAs) and monounsaturated fatty acids (MUFAs), is an ubiquitous metabolic event to sustain the increasing demand of new membrane phospholipids with appropriate acyl composition during tumor development^1^. As the biosynthesis source of various lipids, e.g., triglycerides, diacylglycerols, cholesterol esters, and phospholipids, fatty acids play the important roles in cellular signaling transduction and engage cell bio-function including apoptosis^2^,^3^, lipotoxicity^4^, migration^5^, endoplasmatic reticulum (ER) stress^6^,^7^, differentiation and proliferation^8^,^9^,^10^, which are controlled by the desaturation balance of acyl composition of fatty acids. Therefore, the process of desaturation degree of fatty acids results in cell survival or proliferation during tumor development.

Stearoyl-CoA desaturase-1 (SCD1), a transmembrane protein mainly located at ER organelle, catalyzes SFAs to ∆-9 MUFAs, e.g., converting palmitic acid (C16:0 FA) to palmitoleic acid (C16:1 FA) or converting stearic acid (C18:0 FA) to oleic acid (C18:1 FA)^11^. SCD1 is necessary to stimulate lipid biosynthesis to supply new phospholipids for cell membrane biogenesis in cell cycle process of mitosis^12^. Last decade, SCD1 has been widely studied on cancer research and considered to be a novel molecular target for broad-spectrum tumors^13^,^14^,^15^. Reduction of SCD1 activity and mRNA expression impaired the formation of cell membrane lipids with the decrease of fatty acid biosynthesis and desaturation^13^,^16^, leading to cease cancer cell proliferation and induce cell apoptosis.

The increasing studies of fatty acids on the effect of tumorigenesis have shown that SFA palmitate induces cell apoptosis, promotes monocyte atherogenicity and resists insulin signal transduction through the induction of cellular ceramides levels^17^. Ceramides are the important lipid messages involved in tumor development and progression^18^, and a lower total-ceramide level has been found in tumor tissues^19^. It is composed of sphingosine joined by a fatty acyl base with varying carbon chain length and generated by *de novo* synthesis from palmitoyl-CoA and L-serine^18^. The majority of endogenous ceramides are C16 to C24 ceramides, however, the direct correlation between biological functions and fatty acyl structures of ceramides is still unclear so far. The evidences showed that C16 ceramide involved in stimulating the growth of head and neck squamous cell carcinoma^20^ and C18 ceramide participated in inhibiting cancer cell proliferation^21^.

So, it is intriguing to raise a question: what is the linkage between cellular ceramide signals and SCD1 pathway? Our studies demonstrated that the inhibition of SCD1 activity caused the increase of endogenous cellular SFA levels in both colorectal and ovarian cancer cells, while the increased ceramide levels could be observed only in colorectal cancer cells accompanying with the suppression of cell proliferation. Our further findings elucidated that endo-ceramide biosynthesis was required for SCD1-mediated apoptosis in colorectal cancer.

## Results

### Alternation of SCD1 expression with ceramide signals in colorectal carcinoma patients

**Figure 1:**
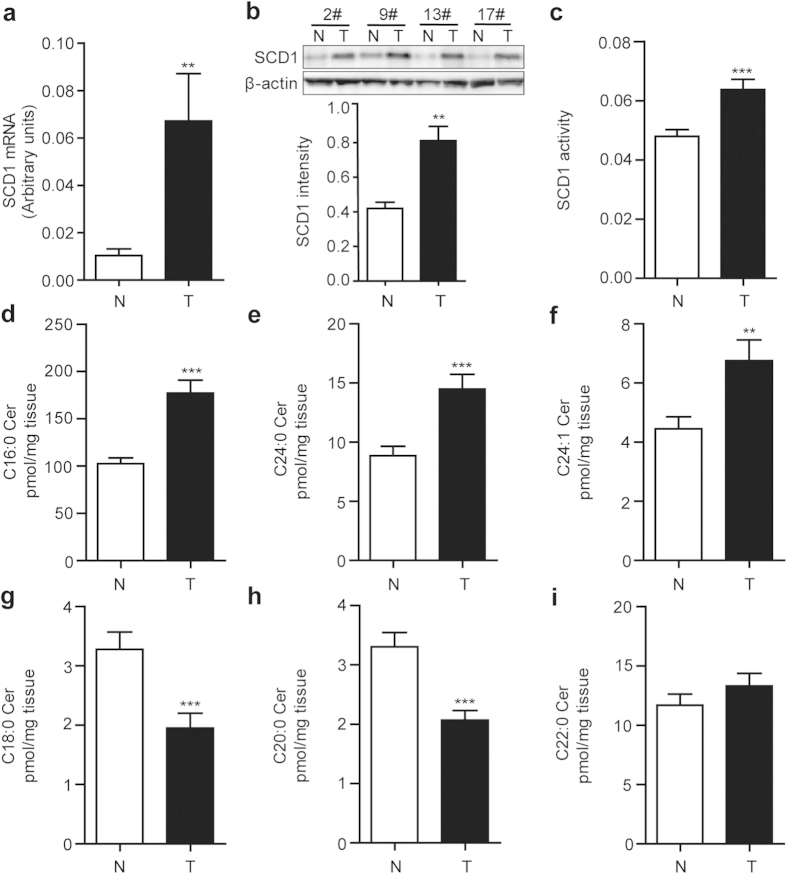
Alteration of SCD1 expression and ceramide signals in human colorectal tumor samples. The levels of (**a**) SCD 1 mRNA expression, (**b**) SCD1 protein expression, density quantification by western-blot, and (**c**) SCD1 activity were significantly elevated in colorectal tumor tissues (T) compared to the adjacent non-tumor tissues (N). (**d**-**i**), The contents of endo-ceramides with various fatty acyl chain in colorectal tumor tissues (T) and adjacent non-tumor tissues (N): (**d**) C16:0 ceramide, (**e**) C24:0 ceramide, (**f**) C24:1 ceramide, (**g**) C18:0 ceramide, (**h**) C20:0 ceramide, and (**i**) C22:0 ceramide. ^**^P < 0.01, ^***^P < 0.001 *vs* N group, Student’s *t*-test, *n* = 45.

To gain the information of SCD1 and endo-ceramide signals in tumor development, we analyzed the expression levels of them in tumor tissues obtained from colorectal cancer patients. The quantitative real-time PCR (Q-PCR) analysis showed that mRNA expression levels of SCD1 in tumor tissues markedly elevated compared to that in adjacent non-tumor tissues (Fig. 1a). Consistent with mRNA expression, the protein expression and enzymatic activity of SCD1, accessed by western-blot and ratio of C16:1 fatty acid to C16:0 fatty acid contents, were found to increase in colorectal tumor tissues (Fig. 1b,c).

Total 90 samples of colorectal tumor tissues and the matched adjacent non-tumor tissues were examined by HPLC-MS/MS analysis. The alternation of endoceramides profile was observed, increasing the levels of C16:0 ceramide, C24:0 ceramide, C24:1 ceramide (Fig. 1d–f) and decreasing the levels of C18:0 ceramide and C20:0 ceramide (Fig. 1g,h), while unchanging the levels of C22:0 ceramide (Fig. 1i).

### Blockage of SCD1 suppresses cell proliferation via cellular ceramide signals

**Figure 2:**
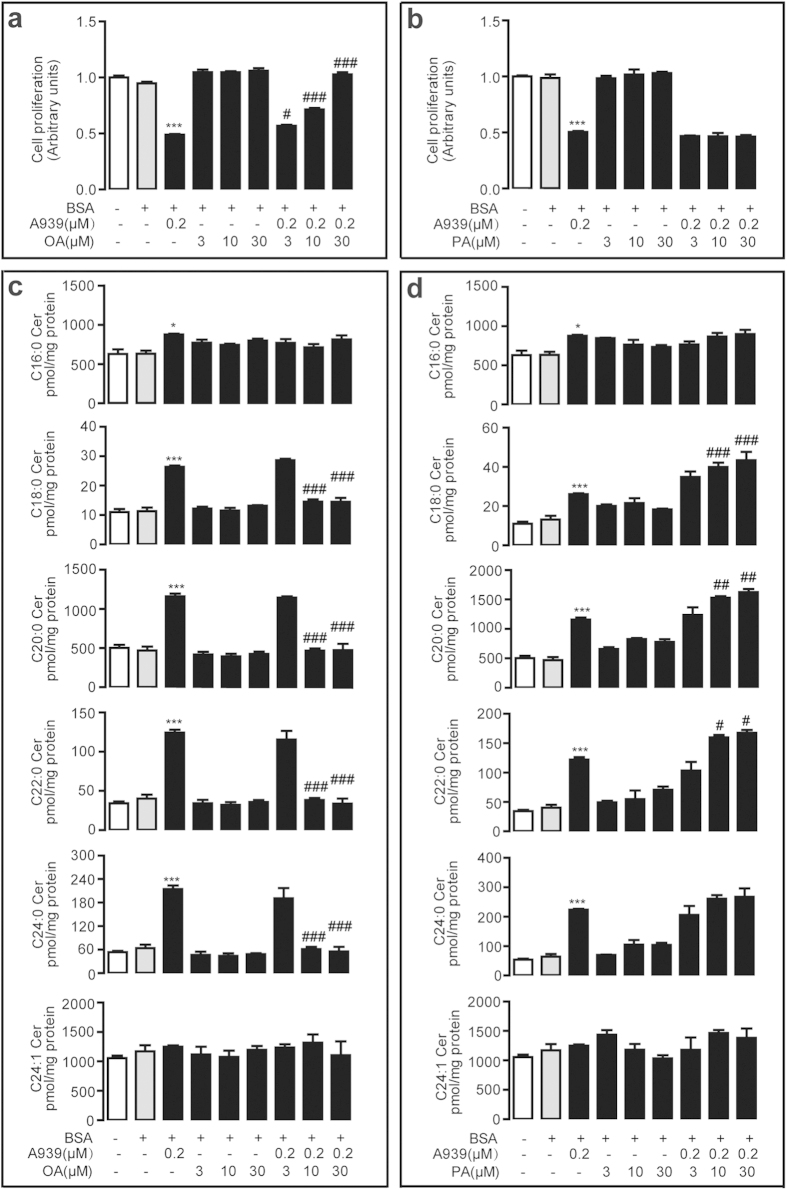
A939-inhibited cell proliferation was reversed by Oleate. (**a**,**b**) The dose-dependent effect of oleic acid (OA) (**a**) and palmitic acid (PA) (**b**) in 0.2 μM A939-inhibited cell proliferation in LOVO cells. (**c**,**d**) The dose-dependent effect of oleic acid (OA) (**c**) and palmitic acid (PA) (**d**) in 0.2 μM A939-induced endo-ceramide levels in LOVO cells. ^*^P < 0.05, ^***^P < 0.001 *vs* vehicle group; ^#^*P* < 0.05, ^##^*P* < 0.01, ^###^*P* < 0.001 *vs* 0.2 μM A939 group, one-way ANOVA followed by Bonferroni compare all pairs test, *n* = 4.

SCD1 has been considered as a novel therapeutic target for renal cell carcinoma^15^. Here, we investigated the effect of SCD1 on tumor cell proliferation by blocking SCD1 activity with specific SCD1 inhibitors A939572 (A939) and Cay10566 (Cay105) (Supplementary Fig. 1). In human colorectal adenocarcinoma LOVO cells, 0.2 μM A939 significantly inhibited cell proliferation and this effect could be dose-dependently reversed by oleic acid (Fig. 2a), but not by palmitic acid (Fig. 2b). The HPLC-MS/MS analysis found that 0.2 μM A939 dramatically increased intracellular long-chain saturated ceramide levels including C18:0 ceramide, C20:0 ceramide, C22:0 ceramide, and C24:0 ceramide, while it slightly increased middle-chain saturated ceramide C16:0 and not affected mono-unsaturated C24:1 ceramide (Fig. 2c,d). Oleic acid (Fig. 2c), but not palmitic acid (Fig. 2d), effectively eliminated SCD1 inhibitor-induced cellular ceramide (C18:0, C20:0, C22:0, C24:0) production in human LOVO cells.

**Figure 3:**
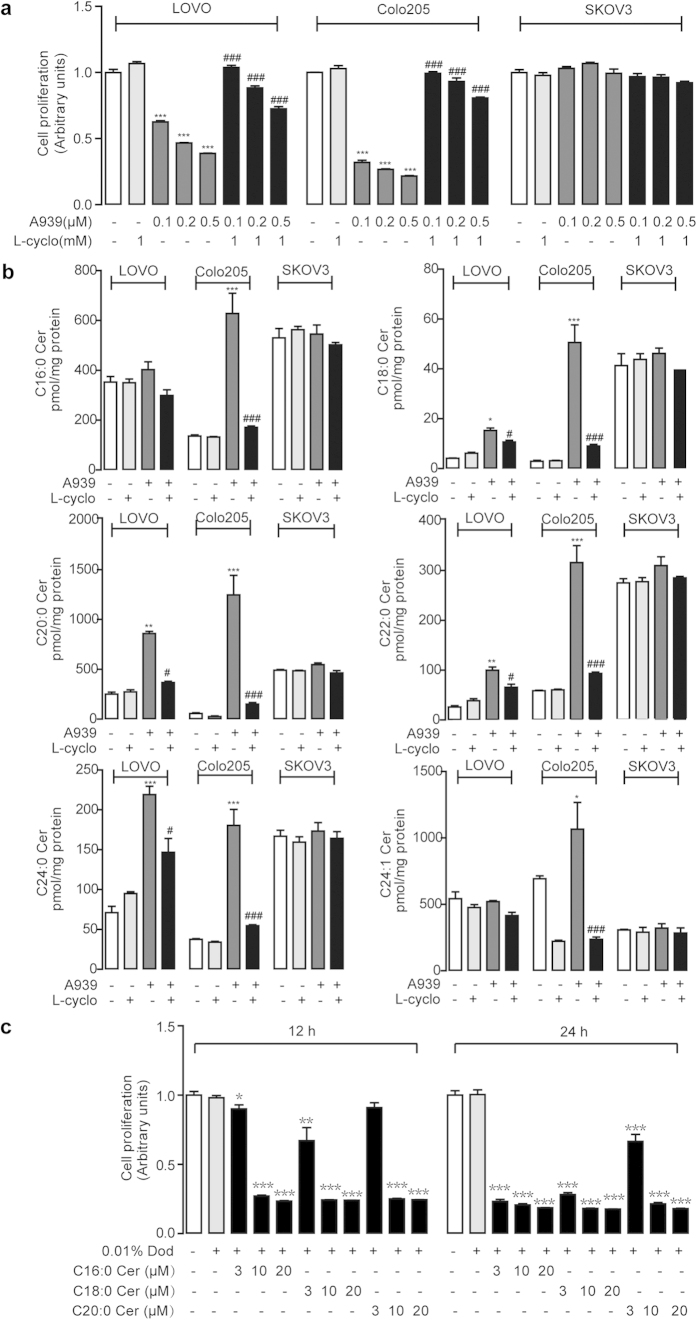
A939 suppressed cell proliferation through endo-ceramide signals. (**a**) The effect of dose-dependent A939 without or with L-cyclo (1 mM) on cell proliferation in human colorectal cancer, LOVO cells and Colo205 cells, and human ovarian cancer SKOV3 cells. (**b**) The effect of A939 (0.2 μM) without or with L-cyclo (1 mM) on cellular endo-ceramide contents, accessed with HPLC/MS-MS, in LOVO cells, Colo205 cells, and SKOV3 cells. (**c**) The effect of C16:0 ceramide, C18:0 ceramide, or C20:0 ceramide on cell proliferation in LOVO cells in dose-dependent and time-dependent manners. Vehicle, 0.01% dodecane (Dod). ^*^P < 0.05, ^**^P < 0.01, ^***^P < 0.001 *vs* vehicle group, and ^#^*P* < 0.05, ^###^*P* < 0.001 *vs* A939 group, One-way ANOVA analysis, *n* = 4.

To further understand the correlation of SCD1-mediated cell proliferation and endo-ceramide signals, we detected cell proliferation and ceramide contents in various tumor cell lines, including human colorectal adenocarcinoma cells, LOVO and Colo205, and human ovary adenocarcinoma SKOV3 cells, after treating with A939. A939 dose-dependently suppressed cell proliferation in LOVO cells and Colo205 cells, but not in SKOV3 cells (Fig. 3a). However, A939-induced cell proliferation in LOVO cells and Colo205 cells was eliminated by 1 mM L-cycloserine (L-cyclo) or 10 μM myriocin (Fig. 3a and Supplementary Fig. 2), two specific inhibitors of serine palmitoyltransferase (SPT) involved in ceramide biosynthesis.

Although SCD1 activity were inhibited after A939 treatment in both colorectal and ovarian cancer cells (Supplementary Fig. 1), the examination of ceramides contents showed that A939 induced the ceramide production in LOVO cells and Colo205 cells, but not in SKOV3 cells (Fig. 3b). Simultaneously, L-cyclo significantly reversed A939-induced endo-ceramides elevation (Fig. 3b). In addition, another potent SCD1 blocker Cay105 was used to further confirm the effects, showing that the inhibition of cell proliferation along with the increase of cellular ceramide signals in LOVO cells (Supplementary Fig. 3a and Supplementary Fig. 4) and Colo205 cells (Supplementary Fig. 3b and Supplementary Fig. 5), but not in SKOV3 cells (Supplementary Fig. 3c and Supplementary Fig. 6), were observed the same as A939. Moreover, mice vascular smooth muscle cells (VSMCs) were also used to evaluate proliferation effects of SCD1 inhibition. A939 also compressed proliferation of VSMCs (Supplementary Fig. 7).

RNA interference was also applied to further confirm the crosstalk between SCD1 blockage and ceramide signals. SiRNA transfection significantly inhibited the protein and mRNA expression level of SCD1 in LOVO cells (Supplementary Fig. 8a,b). In addition, cell proliferation was also suppressed by RNAi, which was also reversed by L-cyclo (1 mM) (Supplementary Fig. 8c). Result of simultaneous detection of cellular ceramides was similar to the one by SCD1 inhibitor treatment (Supplementary Fig. 8d).

To test whether the modulation of cell proliferation accounted for the elevation of ceramide expression, we treated human LOVO cells with exogenous ceramides and found that C16:0 ceramide, C18:0 ceramide, and C20:0 ceramide significantly suppressed cell proliferation respectively in dose-dependent and time-dependent manners (Fig. 3c).

### Ceramide mediated cell apoptosis induced by SCD1 Blockage

**Figure 4:**
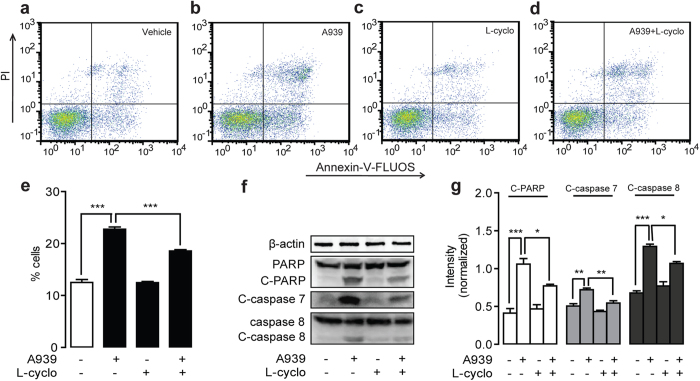
L-cyclo reversed A939-induced cell apoptosis in human colorectal cancer LOVO cells. (**a–d**) The effect of vehicle (**a**), 0.2 μM A939 (**b**), 1 mM L-cyclo (**c**), and 0.2 μM A939 combined with 1 mM L-cyclo (**d**) on cell apoptosis, determined with Annexin-V-FLUOS/PI by FACS. (**e**) Quantitative analysis of cell apoptosis from data of panels (**a-d)**. (**f**,**g**) The effect of 0.2 μM A939 without/with 1 mM L-cyclo on the protein expression levels of PARP, cleaved-PARP, cleaved-caspase 7, caspase 8 and cleaved-caspase 8, accessed by western-blot (**f**) and quantification of protein expression levels normalized with *β*-actin (**g**). ^*^P < 0.05, ^**^P < 0.01. ^***^P < 0.001, One-way ANOVA analysis, *n* = 3.

Next, we analyzed the cell cycle profile of SCD1-mediated inhibitory cell proliferation using Annexin-V-FLUOS/PI kit. Flow cytometer analysis showed that, comparing to vehicle treatment (Fig. 4a and Supplementary Fig. 9a), 0.2 μM A939 markedly induced cell apoptosis in LOVO cells (Fig. 4b) and Colo205 cells (Supplementary Fig. 9b), which were significantly blocked by 1 mM L-cyclo (Fig. 4d,e and Supplementary Fig. 9d,e). Western-blot analysis showed that the expression levels of cleaved form of PARP, cleaved form of caspase 7 and cleaved form of caspase 8, the key apoptosis-related proteins, but not PARP and caspase 8, were elevated with 0.2 μM A939 administration in LOVO cells (Fig. 4f,g) and Colo205 cells (Supplementary Fig. 9f,g), and these changes were also blocked by 1 mM L-cyclo (Fig. 4f,g and Supplementary Fig. 9f,g).

**Figure 5:**
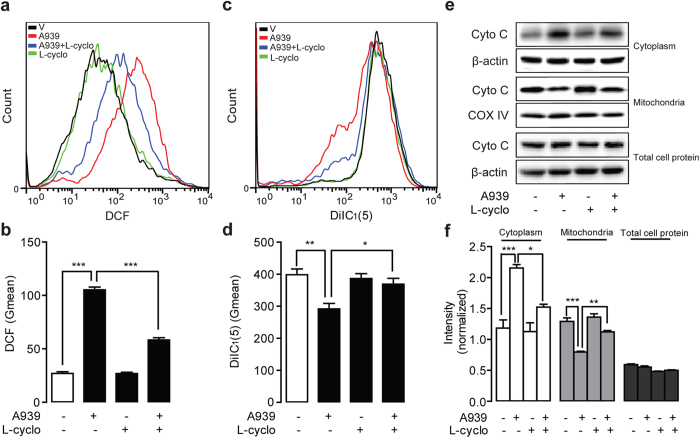
L-cyclo alleviated A939-induced mitochondria dysfunction in LOVO cells. The effect of 0.2 μM A939 without/with 1 mM L-cyclo on (**a**,**b**) cellular ROS production, detected with DCF fluorescence in flow cytometer (**a**) and quantification of ROS release with geometrical mean values (Gmean) (**b**); (**c**,**d**) mitochondrial membrane potential, detected with DiIC_1_(5) dye with flow cytometer (**c**) and density quantification (**d**); and (**e**,**f**) cytochrome C protein contents in cytoplasm fraction, mitochondrial fraction, and entire cell, detected by western-blot (**e**) and quantitative analysis by normalizing with *β*–actin or COX IV (**f**). ^*^P < 0.05, ^**^P < 0.01, ^***^P < 0.001, One-way ANOVA analysis, *n* = 3.

Mitochondria dysfunction is primary action to participate cell apoptosis process. To explore whether mitochondrial pathway applied to ceramide-mediated apoptosis in colorectal cancer cells, we determined ROS release and mitochondrial transmembrane potential (Δψ_m_) in human LOVO cells and Colo205 cells by flow cytometer. Flow cytometer analysis found that ROS generation was induced by A939, Cay105 and SCD1 siRNA, respectively in LOVO or Colo205 cells (Fig. 5a,b, Supplementary Fig. 10a,b and Supplementary Fig. 11). At the same time, 0.2 μM A939 could reduce cell mitochondrial potential Δψ_m_ (Fig. 5c,d and Supplementary Fig. 10c,d), accessed with DilC_1_(5), in both the above cells. A939-induced mitochondria dysfunction was rescued when cells were treated with 1 mM L-cyclo (Fig. 5 and Supplementary Fig. 10). As cytochrome C translocation has been considered as an indicator for mitochondria dysfunction, we determined the cytochrome C contents in various cell fractions of LOVO cells and Colo205 cells after SCD1 inhibitors treatment. Western-blot analysis revealed that 0.2 μM A939 treatment increased cytochrome C protein levels in cellular cytoplasm fractions, but decreased cytochrome C protein levels in mitochondrial fractions, while there was no changes in the total amount of cytochrome C protein in human LOVO cells (Fig. 5e,f) and Colo205 cells (Supplementary Fig. 10e,f). A939-induced cytochrome C translocation from mitochondria to cytoplasm was corrected by 1 mM L-cyclo (Fig. 5e,f and Supplementary Fig. 10e,f).

### Inhibition of ceramide *de novo* synthesis reversed tumor shrinkage induced by SCD1 inhibition

**Figure 6:**
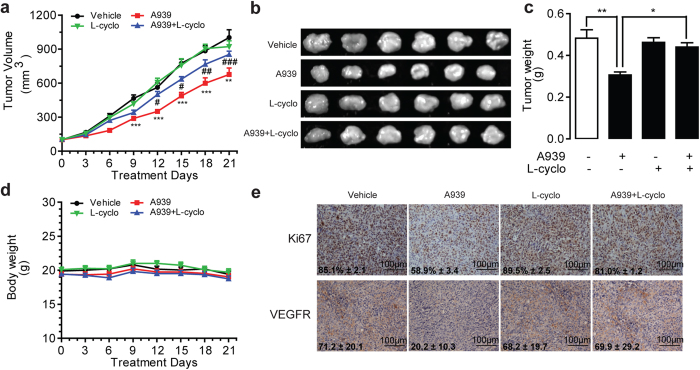
A939 significantly arrests tumor growth in LOVO cell xenograft mice. The effect of vehicle (Tween-80/PEG-400/saline, 5/5/90, i.p.), A939 (10 mg kg^−1^ d^−1^, i.p) without/with L-cyclo (10 mg kg^−1^ d^−1^, i.p.) on (**a**,**b**) tumor volume, (**c**) tumor weight, (**d**) body weight of animals, and (**e**) the expression levels of Ki67 and VEGFR, accessed by immuno-histochemistry analysis. ^*^P < 0.05, ^**^P < 0.01, ^***^P < 0.001 *vs* vehicle group, ^#^*P* < 0.05, ^##^*P* < 0.01, ^###^*P* < 0.001 *vs* A939 group, *n* = 6. Data in panel (**a**) was analyzed by Two-way ANOVA and panel (**c**) was analyzed by One-way ANOVA analysis.

To evaluate the effect of ceramide in SCD1 blockage induced tumor shrinkage, we administrated SCD1 inhibitors with or without L-cyclo in the xenograft tumor mice, subcutaneously injected with LOVO cells. When tumors volume was reached around 100 mm^3^, A939 (10 mg kg^−1^ d^−1^, i.p.), with or without L-cyclo (10 mg kg^−1^ d^−1^, i.p.) were administrated for 21 days. Comparing to vehicle administered group, A939 administration, but not L-cyclo administration, significantly suppressed tumor volume and tumor weight (Fig. 6a–c). As predicted, A939-delayed tumor growth was, at least partially, but significantly, reversed by L-cyclo (10 mg kg^−1^, i.p.) (Fig. 6a–c). Notably, no body weight changes were observed in all groups of drug regimens (Fig. 6d) and anti-tumor effect by A939 even started to happen after 9 days drug regimen (Fig. 6a). Concomitant with tumor characteristics, immunohistochemistry analysis demonstrated that 10 mg kg^−1^ A939, but not L-cyclo, reduced the expression levels of Ki67 and VEGFR, two major tumorigenesis biomarkers, in tumor tissues (Fig. 6e). However, the combination of A939 and L-cyclo eliminated the reduction of Ki67 and VEGFR expression in tumor tissues (Fig. 6e).

## Discussion

As a key conversion enzyme for mono-desaturation of fatty acids, SCD1 regulates lipogenesis event to provide essential lipid resources for rapid proliferation and cell structure in cancer cells. Consistent with previous reports, our data showed an increase of SCD1 mRNA, protein and activity expressions in tumor tissues of colorectal cancer patients (Fig. 1a–c), which suggested that inhibition of SCD1 may be a promising strategy for colorectal cancer control. The mechanism how SCD1 inhibition blocks cancer progression has attracted attention. SCD1 inhibition impairs proliferation of cancer cells by down-regulating the rate of glucose-mediated lipogenesis especially SFA synthesis via AMPK pathway^22^. Ablation of SCD1 expression was also reported to control cancer cell proliferation by decrease Akt phosphorylation or even modulation of glycogen synthase kinase-3 beta, the downstream target of Akt^23^,^24^. In this study, we found that SCD1 inhibitors, A939 and Cay105, restricted colorectal cell proliferation along with a predominant up-regulation of cellular ceramide contents. Although slightly controversial argument of pathologic roles of ceramides with different carbon chains of fatty acyl, the studies focused on pharmacological function of ceramides on cancer research still get the attention.

Both SCD1 inhibition and ceramide signals have shown the pharmacologic properties in the regulation of tumor development. We assumed that cellular endogenous ceramide signals might mediate the effects of SCD1 inhibition. Roemeling *et al.* reported that supplemental exogenous oleate rescued the inhibition of SCD1-induced cell death in clear cell renal cell cancer and lung cancer cells^15^,^25^. Our data found the similar observation, and further identified that supplemental oleate counteracted the role of SCD1 inhibitor by rescuing the increase of ceramides in LOVO cells (Fig. 2). Further experiment results demonstrated that blocking ceramide synthesis pathway by L-cyclo was able to eliminate the colorectal cancer cell arrest and endo-ceramide production induced by SCD1 inhibitors (Fig. 3, Supplementary Figs 3–5), and another ceramide synthesis inhibitor myriocin also showed similar proliferation rescue ability against SCD1 inhibition (Supplementary Fig. 2), which confirmed the hypothesis. The increasing contents of long-chain ceramides by SCD1 inhibitor contributed to mitochondria dysfunction^26^ including ROS induction, alteration of mitochondria membrane potential, and release of cytochrome C, which leaded to tumor cell apoptosis (Figs 4 and 5). Although, it was suggested that normal cells displaying lower SCD1 activity were resistant to the cytotoxicity of SCD1 inhibition, and we found that proliferation block was induced by A939 in a dose-dependent manner in VSMCs, which was similar with the apoptosis reports on islet cell line MIN6 and L6 myotubes^27^,^28^.

As we demonstrated that SCD1 inhibitors and siRNA both increased the SFA accumulation in human colorectal cancer LOVO and Colo205 cells as well as in human ovarian cancer SKOV3 cells (Supplementary Fig. 1), while they did not induce SKOV3 cell apoptosis along with obvious endo-ceramide production (Fig. 2, Supplementary Figs 3–6). It is well reported that SCD1 inhibition causes cancer cell death by SFAs accumulation and MUFAs depletion^29^. Here, we suggested that endogenous ceramides were fundamental substance for SCD1-mediated anticancer property rather than FFAs. SFAs but not MUFAs were supposed to induce the formation of sphingolipids including ceramides and glucosylceramides. It is reported that endogenously produced ceramides are necessary for the development of SCD1 inhibition-induced cell apoptosis related to insulin-resistant^30^. The elevated SFA might activate the TLR4 cascade to increase rates of *de novo* ceramide synthesis. As far as we know now, the expression and signaling of TLR4 pathway in SKOV3 is not abnormal^31^, so it is hard to explain this different result. Howerer, some other research indicated that *de novo* ceramide synthesis was not required for SFAs-induced apoptosis in CHO cells^32^ and H4IIE liver cells^33^. The detailed mechanism by which SCD1 inhibition affects ceramide synthesis has been unknown. Elucidation of the distinctive ceramide biosynthetic pathway in various cells and organs is helpful for disclosing the behind mechanisms.

In summary, high SCD1 level was found in colorectal cancer tissues compared to adjacent normal tissues, suggesting that SCD1 might be used as a predictive biomarker and therapeutic target for colorectal cancer. *In vivo* and *in vitro* pharmacological studies revealed that SCD1 inhibition ceased colorectal tumor growth through the induction of cellular ceramide production, mitochondria dysfunction and apoptosis in colorectal cancer cells. It will be interesting to investigate in the future the mechanism underlying the cross-talk of SCD1 and ceramide synthesis pathway during colorectal cancer development.

## Materials and Methods

### Chemicals

A939572 and Cay10566 were purchased from Abcam (Shanghai, China) and Cayman (Shanghai, China) respectively. L-cycloserine and oleic acid (OA) were obtained from Santa Cruz Biotechnology (CA, USA) and Sangon (Shanghai, China). Internal standards of ceramides and fatty acids were purchased from Avanti (Alabaster, USA). Palmitic acid, Bovine serum albumin (BSA), and other chemical were purchased from Sigma-Aldrich (Shanghai, China).

### Patients and tissue preparation

Tumor tissues were obtained from colorectal cancer patients undergone surgical removal at the Department of Colorectal Surgery in Fujian Medical University Union Hospital (Fuzhou, China). All of 45 patients were diagnosed as colorectal cancer according pathology analysis. Non-tumor control tissues were resected from region of 5–7 cm upper the tumor-site from the same patient. After resection, tissue samples were immediately frozen in liquid nitrogen and then stored in an −80 °C freezer for following determination. Informed consents were obtained before surgery from all patients. Scientific study of human subject was approved by the ethics committee in Fujian Medical University Union Hospital following clinical registration guideline in China.

### Cell culture

Colorectal cancer cells LOVO and Colo205 were obtained from ATCC (Rockville, USA) and cultured in Dulbecco’s modified essential medium (DMEM) (Invitrogen, Shanghai, China) supplemented with 10% Fetal Bovine Serum (FBS) (Invitrogen). Ovarian cancer cell SKOV3 was a gift of Dr. Y.B. Mao, and cultured in RPMI-1640 medium with 10% FBS at 37 °C under 5% CO_2_. VSMCs was obtained from Clinical Research Institute, The First Affiliated Hospital, University of South China, and cultured in DMEM with 10% FBS.

### RNA isolation and real-time quantitative-PCR

RNA isolation, reverse transcription, and real-time quantitative-PCR (RT-Q-PCR) were conducted as previously described^34^. Briefly, 1 μg of total RNA extracted from tissues or cells was used to synthesize cDNA, and then RT-Q-PCR was performed in a 7300 real-time PCR System (Applied Biosystems, CA, USA) using SYBR Premix Ex Taq ™ II TliRnaseH Plus (Takara, Dalian, China). The expression levels of mRNA were normalized by house-keeping gene ribosomal RNA 18S. Primers of the target genes were as follows: SCD1, forward primer (F): CACCCAGCTGTCAAAGAGAAGG, reverse primer (R): AGGACGATATCCGAAGAGGTGG; 18S, F: CAGCCACCCGAGATTGAGCA, R: TAGTAGCGACGGGCGGTGTG.

### Lipids extraction and analysis

Lipids were extracted from frozen patient samples as described^35^,^36^ with slight modification^37^. Tissues (20–30 mg) were homogenized, followed by ultrasonication in methanol/water mixture (vol/vol, 1/1, 1 ml) containing two internal standards C17:0 ceramide (100 pmol, internal standard for ceramides) and C17:0 margaric acid (100 pmol, internal standard for fatty acids). The homogenate was extracted with 4 ml chloroform with vortexing for 1 min. Lipid layer was separated from aqueous layer by centrifugation at 3,000 × g for 10 min, then transferred into a clean 10 ml V-bottom glass tube and dried under nitrogen flow (N_2_). 1 ml chloroform was added, dried spot was reconstituted, and solid-phase extraction was eluted by methanol/chloroform mixture (vol/vol, 1/9). The elution containing ceramides and free fatty acids was dried under N_2_, and reconstituted in 100 μl methanol for HPLC-MS/MS analysis. The Q1/Q3ion pairs were detected at 254.7/236.9 for C16:0 fatty acid (FA), 269.0/250.9 for C17:0 FA, 252.9/234.9 for C16:1 FA, 520.4/264.2 for C16:0 ceramide (Cer), 534.3/264.2 for C17:0 Cer, 548.4/264.2 for C18:0 Cer, 576.4/264.2 for C20:0 Cer, 604.5/264.2 for C22:0 Cer, 632.4/264.2 for C24:0 Cer, 630.4/264.2 for C24:1 Cer.

### Preparation of fatty acid-BSA complexes

Fatty acid-BSA complex solution was prepared as described^38^ before experiment. Briefly, fatty acid solution was added dropwise into 10% fatty acid-free BSA with a volume ratio of 1/10 at 55 °C. After vortex for 10 sec, the mixture solution was incubated at 55 °C for additional 10 min, and sterile filtered before using.

### Mitochondria Isolation

Subcellular fractionations from LOVO cell and Colo205 cell were processed using Mitochondria isolation Kit (Pierce, Rockford, IL) following the manufacturer’s instruction. Cells were harvested with scrapping, added with mitochondria isolation reagents, and homogenized by a Dounce Tissue Grinder (Scientz, Ningbo, China) followed by centrifugation at 700 × g for 10 min at 4 °C to discard cell debris. The supernatant was further centrifuged at 3,000 × g for 15 min to obtain the supernatant as cytosol faction, and the pellets as pure mitochondrial fraction.

### Proliferation assay

Effects of A939, L-cyclo, OA-BSA and PA-BSA on cell viability were measured using the Cell Counting Kit-8 (CCK8) (Dojindo Molecular Technologies, Shanghai, China) according to the manufacturer’s instructions. Cells (3,000 cells per well) were seeded in 96-well plate with treatment of drug candidates. After 48 h culture, each well was added with 10 μl tetrazolium reagent provided from the kit, and incubated at 37 °C for 1 h. The plate was measured at an absorbance wavelength of 450 nm using a microplate reader (Beckman, CA, USA). All the experiments were performed in triplicate.

### Western blot assay

Tissue and cell lysates were prepared by homogenated and ultrasonicated with RIPA buffer, separated on 10%–12% SDS/PAGE acrylamide gel, and then transferred on nitrocellulose membranes (Bio-rad, CA, USA). Membranes were blocked with 10% non-fat milk, incubated at 4 °C overnight with the primary antibody against PARP, cleaved-PARP, cleaved-caspase 7, cleaved-caspase 8, caspase 8, SCD1 (Cell signaling technology, MA, USA), cytochrome C (R&D systems, Minnesota, USA), Cox IV (Abcam, Shanghai, China) and β-actin (Proteintech, Wuhan, China). Secondly antibodies (Multisciences, Hangzhou, China) were applied in accordance with primary antibody, and incubated at room temperature for 1 h. After washing with 1 × PBS, protein bands were visualized by an Electrochemiluminescence Plus kit (Millipore, MA, USA), and quantified using Quantity One Software (Bio-Rad).

### Flow cytometry assay

Annexin-V-FLOUS/PI apoptosis kit (Roche, Mannheim, Germany) was used for apoptosis analysis. After drug treatment for 24 h, cells were harvested, re-suspended in Annexin-V-FLOUS/PI solution, incubated at room temperature for 15 min, and then analyzed with flow cytometer (Partec, Munster, Germany). For ROS detection, cells were incubated with DCFH-DA (5 μM) (Beyotime Biotechnology, Shanghai, China), a ROS-sensitive fluorescent probe, at 37 °C for 30 min, and washed with 1 × PBS for three times. Fluorescence release for DCF was detected with flow cytometer. For assessing mitochondrial transmembrane potential (Δψ_m_), we used the MitoProbe^TM^ 1,1′,3,3,3′,3′-hexamethylindodicarbocyanine iodide (DiIC_1_(5)) Assay Kit (Invitrogen) according manufacturer instruction. Cells were stained with DiIC_1_(5) (50 nM) at 37 °C for 30 min, and analyzed with flow cytometer. Data were analyzed with FlowJo 7.6.5 Software.

### Animal Studies

Balb/c nude mice, six weeks old, were obtained from Slac (Shanghai, China). Mice were maintained in cages with free-accessed food and water under a 12 h dark/light cycle and a temperature of 22 ± 3 °C. All studies in mice were approved by the Institutional Animal Care and Use Committee of Xiamen University, China. 5 × 10^6^LOVO cells were subcutaneously injected into the right fore flank of each nude mouse. The daily drug treatment began when tumor size reached ~100 mm^3^ and continued for 3 weeks as following: A939 group, A939 (10 mg kg^−1^ d^−1^, i.p.) dissolved in assisted solvent (Tween-80/Polyethylene glycol 400/saline, 5/5/90, vol/vol/vol); L-cyclo treatment group, L-cyclo (10 mg kg^−1^ d^−1^, i.p.) in saline; Combined treatment group were given with both drugs at the same dose per day; Control groups were given with the assisted solvent same as A939 group. Body weight and tumor volumes were measured every 3 days with a balance or with a vernier caliper, and then the tumor volume was calculated with the formula: 1/2×[ length×(width)^2^ ]. After 3 week treatment, animals were sacrificed with decapitation and tumor tissues were collected for further analysis.

#### Ethics statement

All experiments were carried out in accordance with “Guide and Care and Use of Laboratory Animals” from Narional Institutes of Health (NIH) and approved by the Animal Care and Use Committees of Xiamen University in China. Informed consents were obtained before surgery from all patients. Collection and scientific study of human subject was approved by the ethics committee in Fujian Medical University Union Hospital and Medical College of Xiamen University following clinical registration guideline in China.

### Statistical analysis

Data values were expressed as Mean ± SEM. Statistic significance of two or multiple groups were respectively presented by Student’s *t*-Test or one-way/two-way ANOVA followed by Bonferroni compare all pairs test. Statistical analysis was performed with GraphPad Prism Software (GraphPad Software, CA, USA). *P* < 0.05 was considered as significance.

## Additional Information

**How to cite this article**: Chen, L. *et al.* Stearoyl-CoA desaturase-1 mediated cell apoptosis in colorectal cancer by promoting ceramide synthesis. *Sci. Rep.* **6**, 19665; doi: 10.1038/srep19665 (2016).

## Supplementary Material

Supplementary Information
