# Supplementary material for: Stearoyl-CoA desaturase-1 mediated cell apoptosis in colorectal cancer by promoting ceramide synthesis
Source: Sci Rep. 2016 Jan 27;6:19665. doi: 10.1038/srep19665 (PMC4728559; doi:10.1038/srep19665)
Supplement: Supplementary Information [file srep19665-s1.pdf]

Supplementary Information

Stearoyl-CoA desaturase-1 mediated cell apoptosis in colorectal cancer by promoting ceramide synthesis

Ling Chen, Jie Ren, Longhe Yang, Yanting Li, Jin Fu, Yuhang Li, Yifeng Tian, Funan Qiu, Zuguo Liu, Yan Qiu

Supplementary method

LC-MS/MS method for ceramide detection

Ceramides were detected by the 3200 Q Trap LC-MS/MS system (Applied Biosystems, USA) coupling with an 1100-LC system (Agilent, China). The mobile phase was consisted of methanol and ultra-pure water (pH 7.4), and the gradient elution was as follows: 85% methanol was kept for the first 3 min, followed by a linear gradient from 85% to 100% methanol for 2 min, and then 100% methanol was continued for 15 min. The elution condition turned finally back to 85% methanol at a flow rate of 0.7 mL/min. The column temperature was kept at 40 ℃. Ion detection was monitored by $APCI^{+}$-MRM mode. The detailed MS/MS parameters are described in **Table. S1**, and LC-MS/MS diagrams of ceramides are presented in **Fig. S12**.

Ceramides	C16:0	C18:0	C18:1	C20:0	C22:0	C24:0	C24:1
Precursor ion（m/z）	520.4	548.4	564.4	576.4	604.5	632.4	630.4
Fragment ion（m/z）	264.2	264.2	264.2	262.2	264.2	264.2	264.2
DP（Volt）	85	85	78	76	76	76	76
EP（Volt）	9.5	9.5	9.5	8	8	8	8
CEP（Volt）	15	15	15	7	7	7	7
CE （Volt）	35	35	35	30	30	30	30
CXP（Volt）	3.5	3.5	3.5	2.0	2.0	2.0	2.0

Table. S1. MS/MS parameters for ceramides.

Supplementary Figure 1

Chen et al.

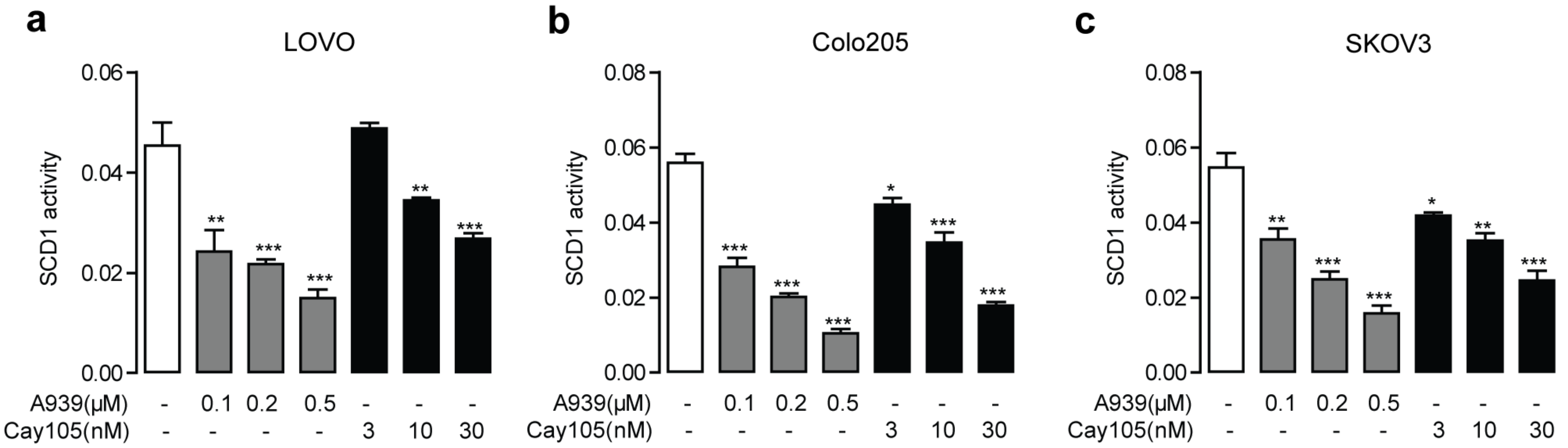

Fig. S1. A939 and Cay105 dose-dependently blocked SCD1 activity of three tumor cell lines. LOVO cells (**a**), Colo205 cellls (**b**) and SKOV3 (**c**) were treated with A939 at three doses (0.1 μM, 0.2 μM, 0.5 μM), or with Cay105 at three doses (3 nM, 10 nM, 30 nM), SCD1 activity was significantly limited. $^{*}P < 0.05$, $^{**} P < 0.01$, $^{***}P < 0.001$ *vs* vehicle group, One-way ANOVA analysis, $n = 4$.

Supplementary Figure 2

Chen et al.

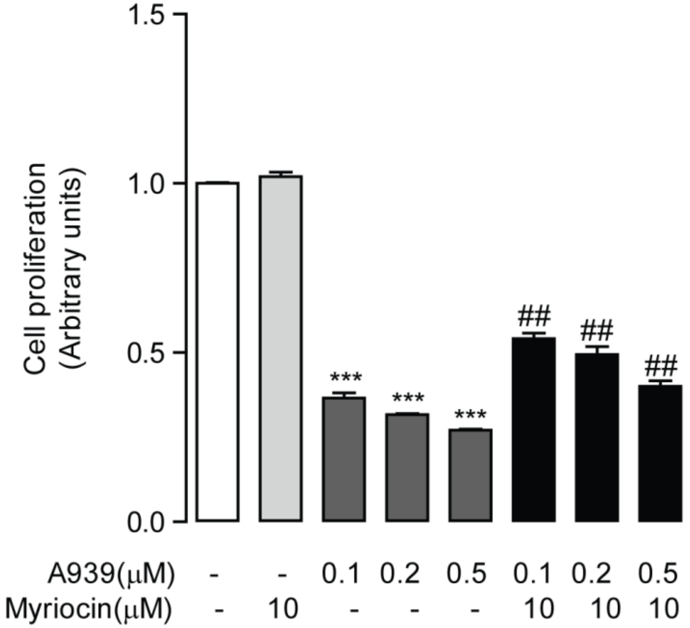

Fig. S2. A939 compressed proliferation of Colo205, which was reversed by ceramide synthesis inhibitor myriocin. A939 decreased proliferation with dose-dependence at 0.1 μM, 0.2 μM and 0.5 μM in Colo205 cells, which was partly counteracted by myriocin (10 μM). $^{***}P < 0.001$ *vs* vehicle group, $^{\#\#}P < 0.01$ *vs* A939 group, One-way ANOVA analysis, $n = 3$.

Supplementary Figure 3

Chen et al.

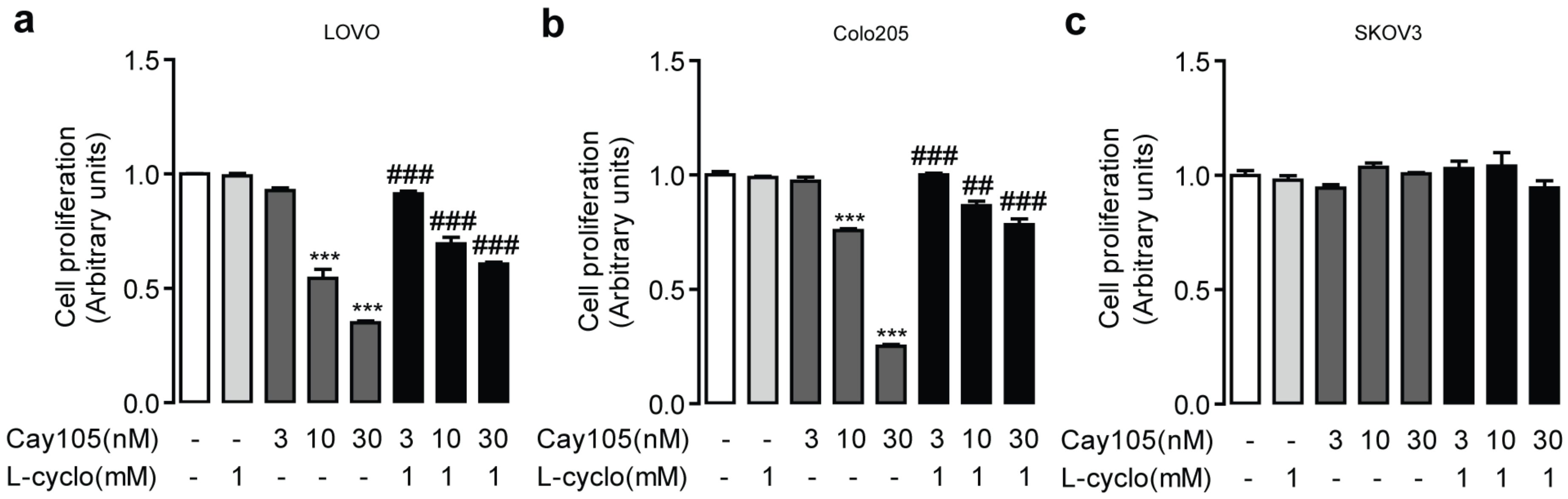

Fig. S3. Cay105 compressed proliferation of LOVO and Colo205, but not SKOV3, which were reversed by L-cyclo. (**a, b**) Cay105 decreased proliferation with dose-dependence at 3 nM, 10 nM and 30 nM, in LOVO cells and Colo205 cells, which were partly counteracted by L-cyclo. (**c**) Cay105 showed no obvious effects on SKOV3 proliferation. $^{***}P < 0.001$ *vs* vehicle group, $^{\#\#}P < 0.01$, $^{\#\#\#}P < 0.001$ *vs* Cay105 group, One-way ANOVA analysis, $n = 4$.

Supplementary Figure 4

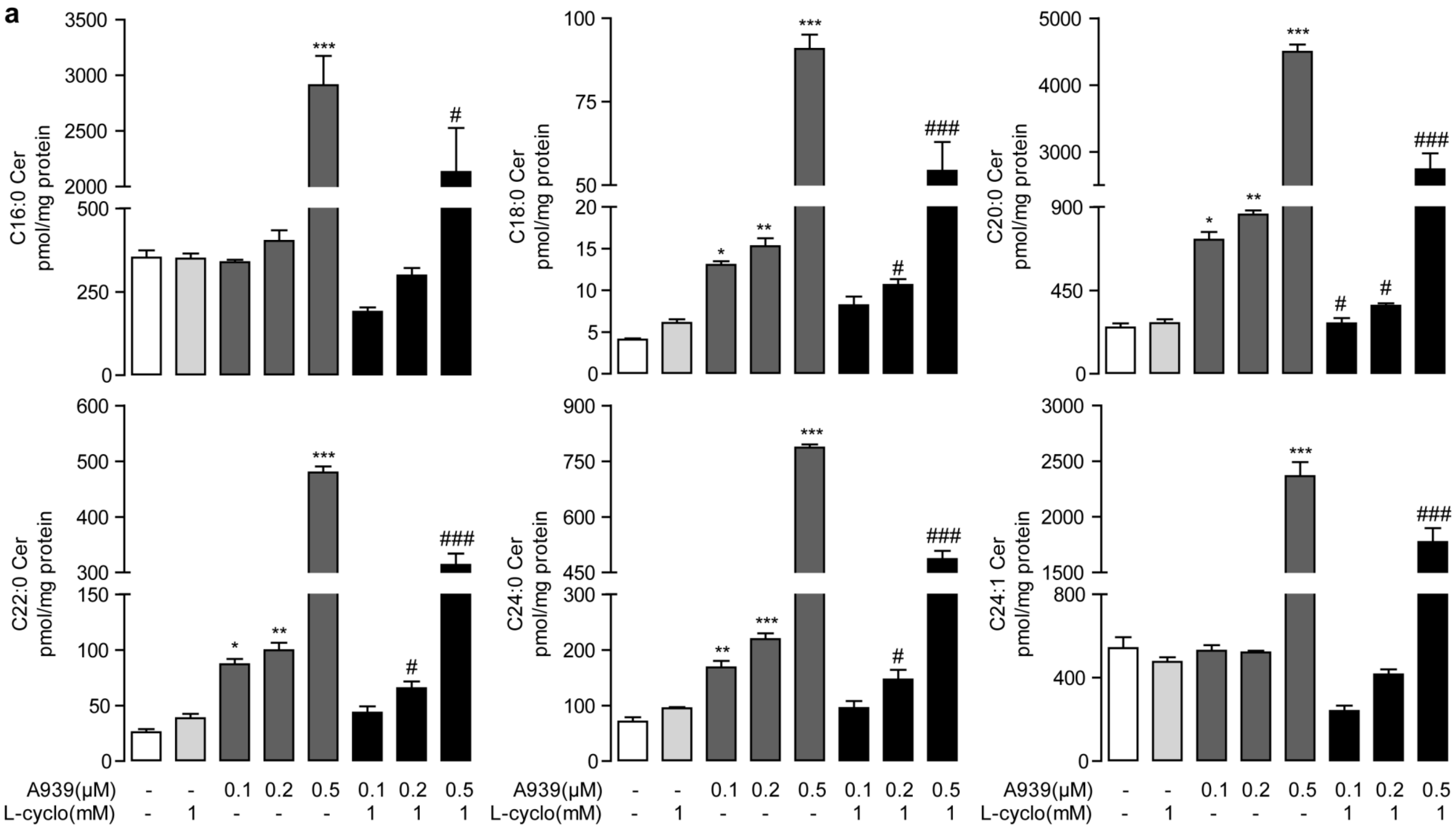

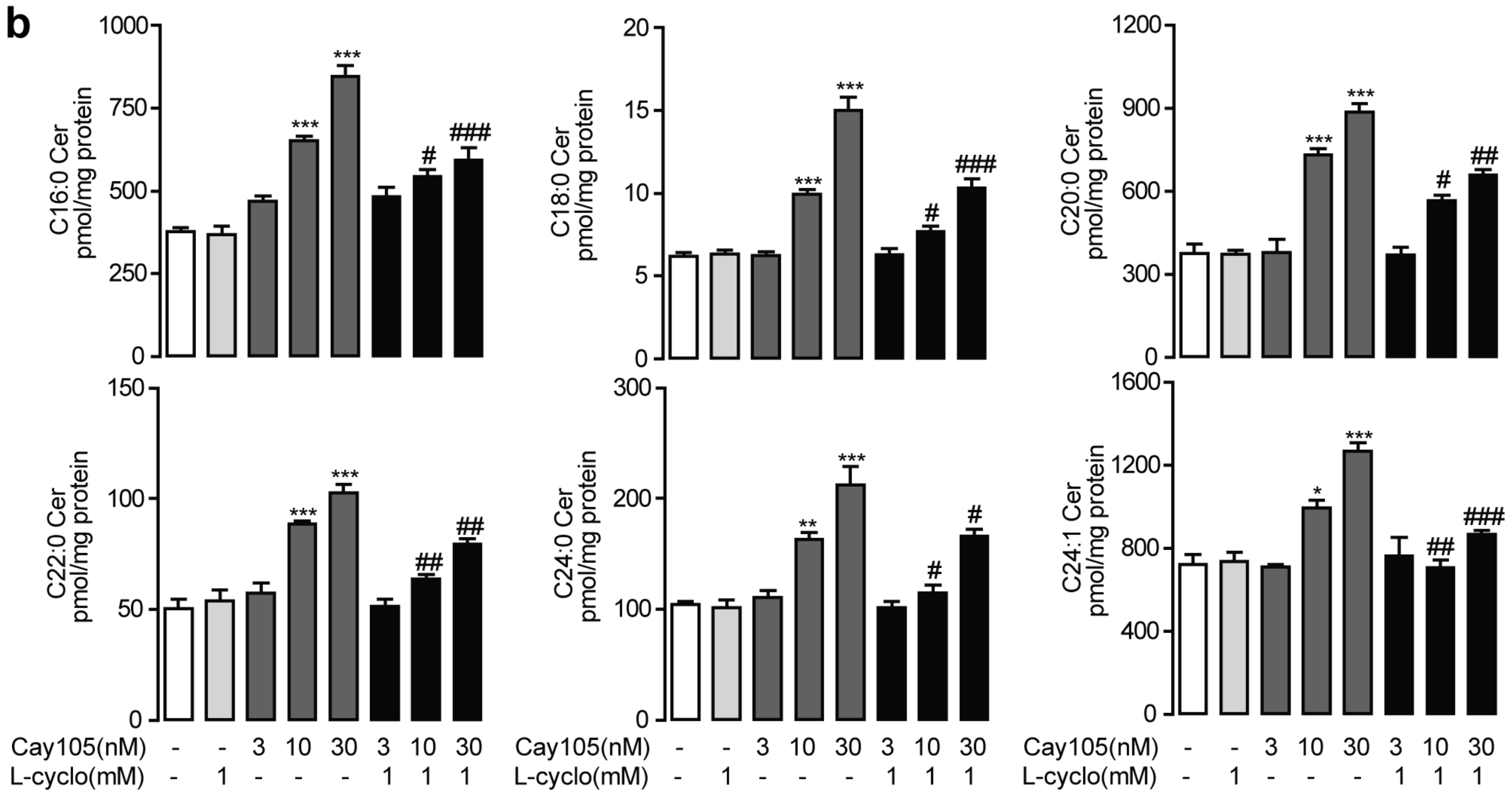

Fig. S4. SCD1 inhibitors elevated ceramide levels of LOVO cells, which were blocked by L-cyclo. (**a**) A939 (0.1 μM, 0.2 μM, 0.5 μM) and (**b**) Cay105 (3 nM, 10 nM and 30 nM) increased endo-ceramide levels of LOVO cells, which were effectively reversed by L-cyclo. $^{*}P < 0.05$, $^{**}P < 0.01$, $^{***}P < 0.001$ *vs* vehicle group, $^{\#}P < 0.05$, $^{\#\#}P < 0.01$, $^{\#\#\#}P < 0.001$ *vs* inhibitor group, One-way ANOVA analysis, $n = 4$.

Supplementary Figure 5

Chen et al.

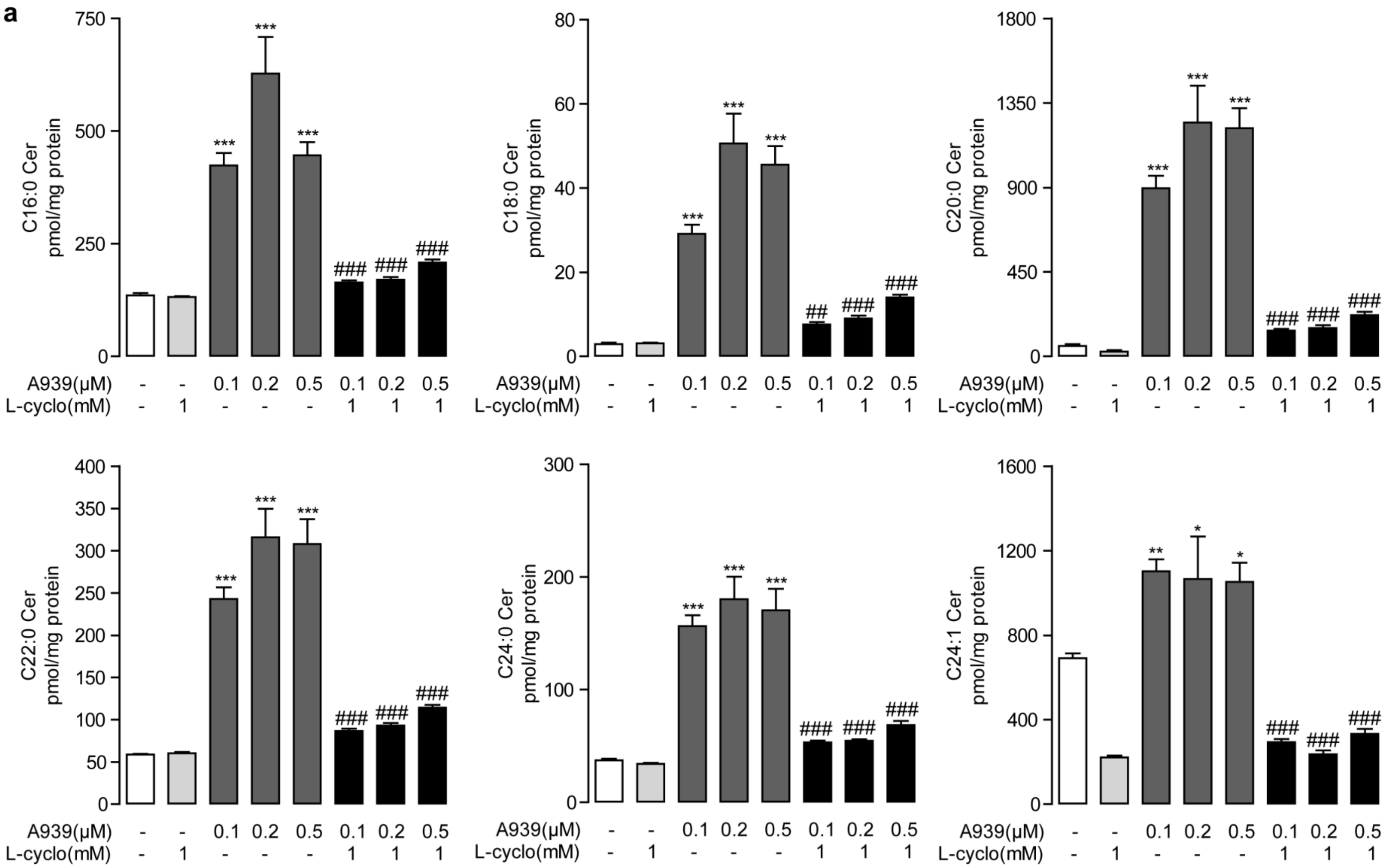

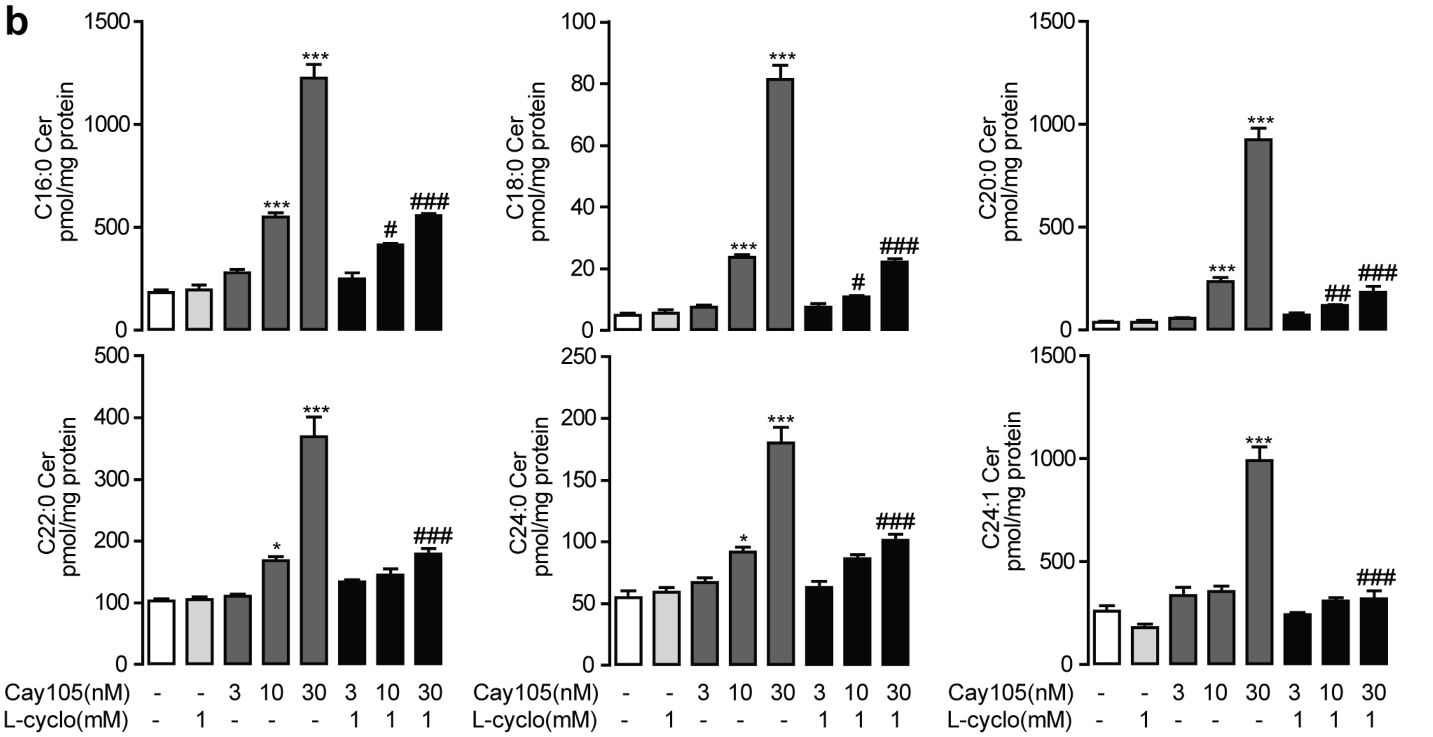

Fig. S5. SCD1 inhibitors elevated endo-ceramide levels in Colo205 cells, which were blocked by L-cyclo. (**a**) A939 (0.1 μM, 0.2 μM, 0.5 μM) and (**b**) Cay105 (3 nM, 10 nM and 30 nM) increased endo-ceramide levels of Colo205 cells, which were effectively reversed by L-cyclo. $^{*}P <$ 0.05, $^{**}P < 0.01$, $^{***}P < 0.001$ *vs* vehicle group, $^{\#}P < 0.05$, $^{\#\#}P < 0.01$, $^{\#\#\#}P < 0.001$ *vs* inhibitor group, One-way ANOVA analysis, $n = 4$.

Supplementary Figure 6

Chen et al.

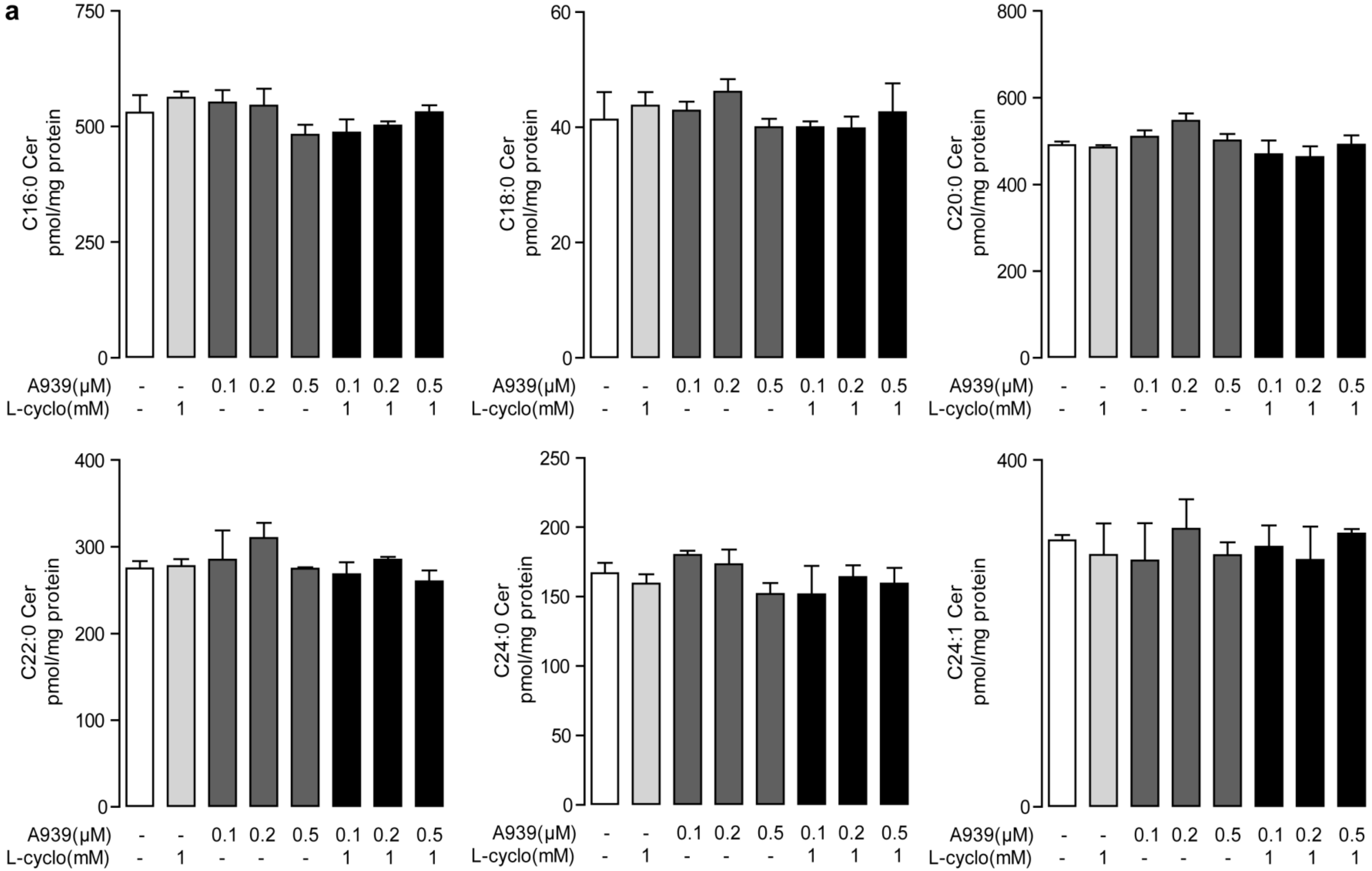

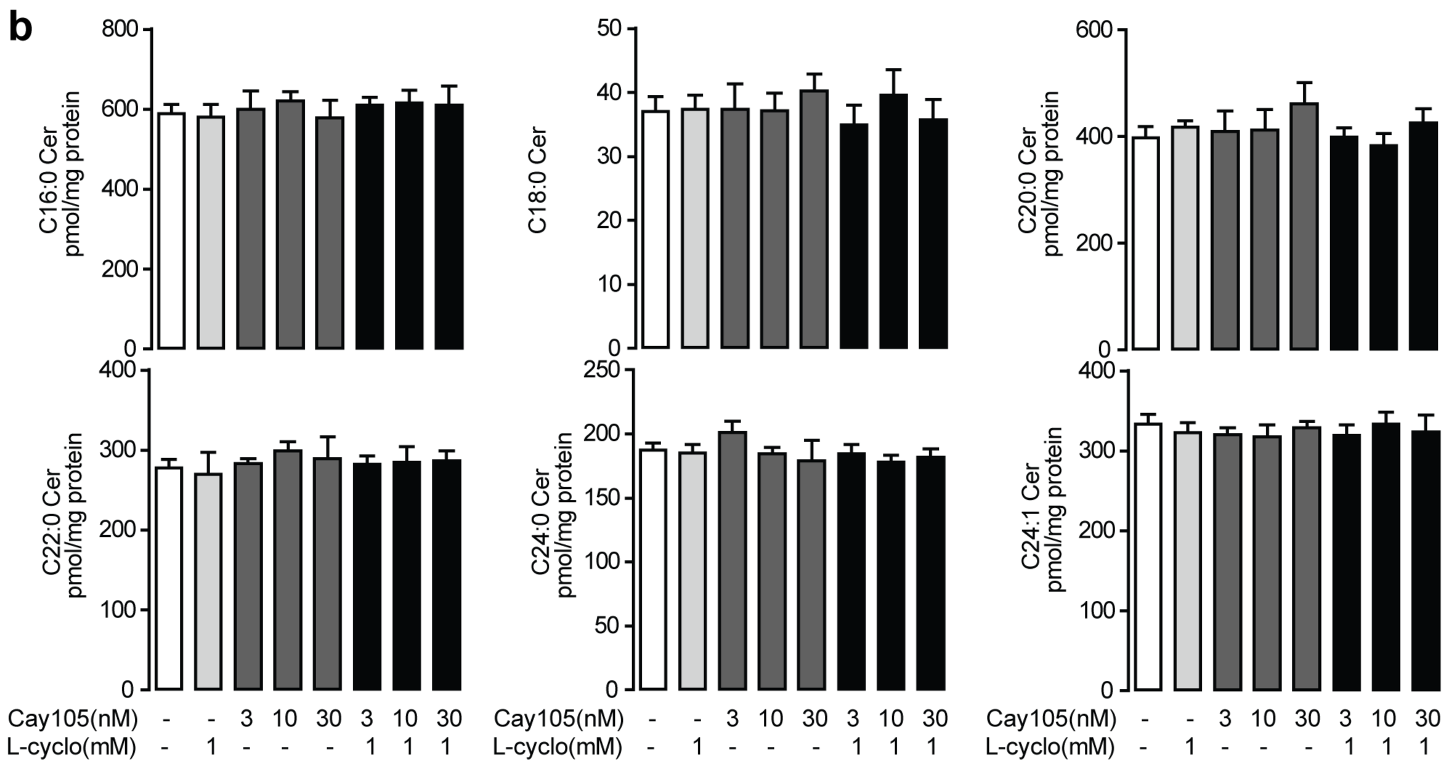

Fig. S6. SCD1 inhibitors showed no effects on endo-ceramide variation in SKOV3 cells. Endo-ceramide levels of SKOV3 cells did not change with administration with (**a**) A939 (0.1 μM, 0.2 μM, 0.5 μM) or (**b**) Cay105 (3 nM, 10 nM and 30 nM).

Supplementary Figure 7

Chen et al.

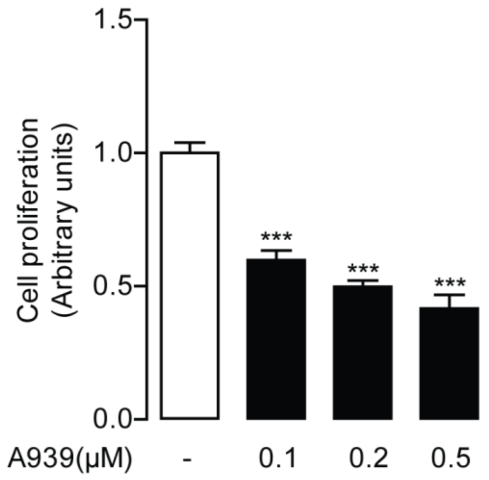

Fig. S7. A939 compressed proliferation of mice vascular smooth muscle cells (VSMCs). A939 decreased proliferation with dose-dependence at 0.1 μM, 0.2 μM and 0.5 μM in VSMCs. $^{***}P < 0.001$ *vs* vehicle group, One-way ANOVA analysis, $n = 3$.

Supplementary Figure 8

Chen et al.

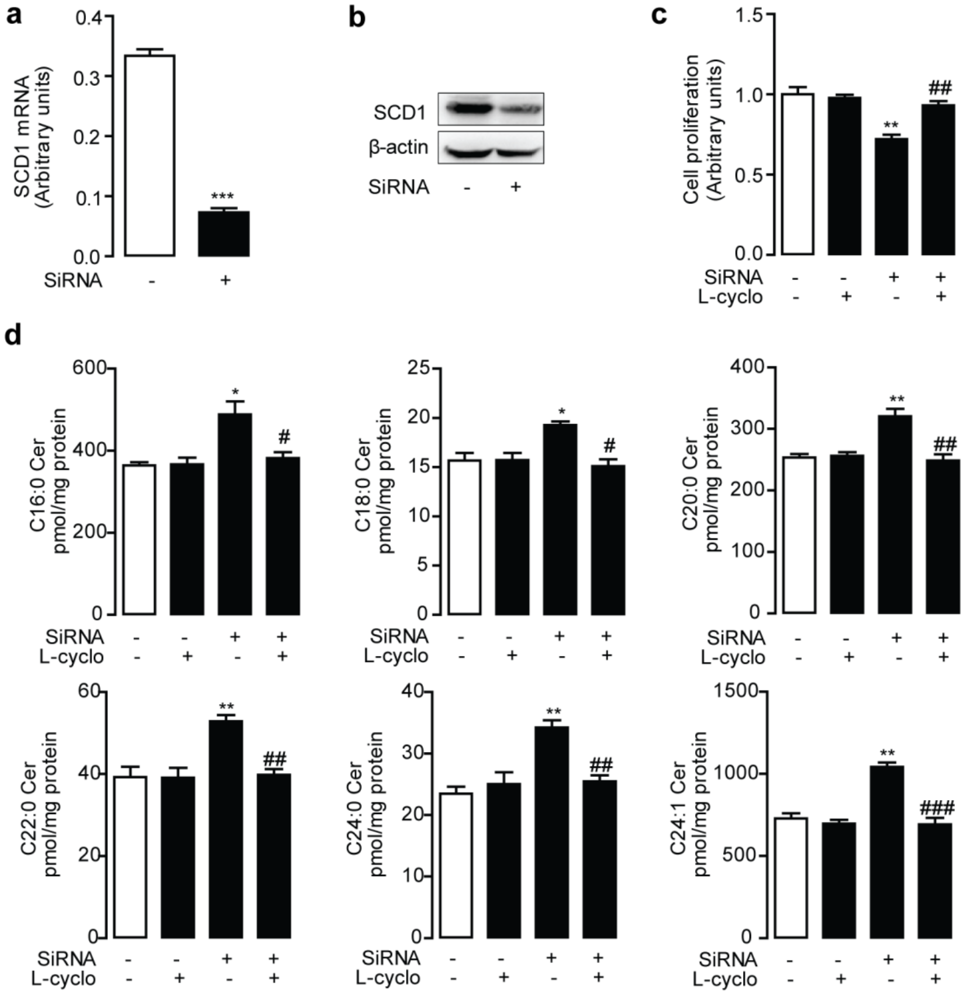

Fig. S8. SCD1 siRNA transfection inhibited cell proliferation and promoted endo-ceramide levels in LOVO cells, which were reversed by L-cyclo. (**a, b**) The mRNA and protein levels of SCD 1 were reduced in LOVO cells when treated with SCD1 siRNA (10 nM) for 48 h. (**c**) SCD1 RNAi suppressed cell proliferation, but was recovered by 48 h treatment of L-cyclo (1 mM). (**d**) SCD1 RNAi increased endo-ceramide levels in LOVO cells, which were reversed by simultaneous L-cyclo treatment. $^{*}P < 0.05$, $^{**}P < 0.01$ *vs* vehicle group, $^{\#}P < 0.05$, $^{\#\#}P < 0.01$, $^{\#\#\#}P < 0.001$ *vs* SiRNA group, One-way ANOVA analysis, $n = 4$.

Supplementary Figure 9

Chen et al.

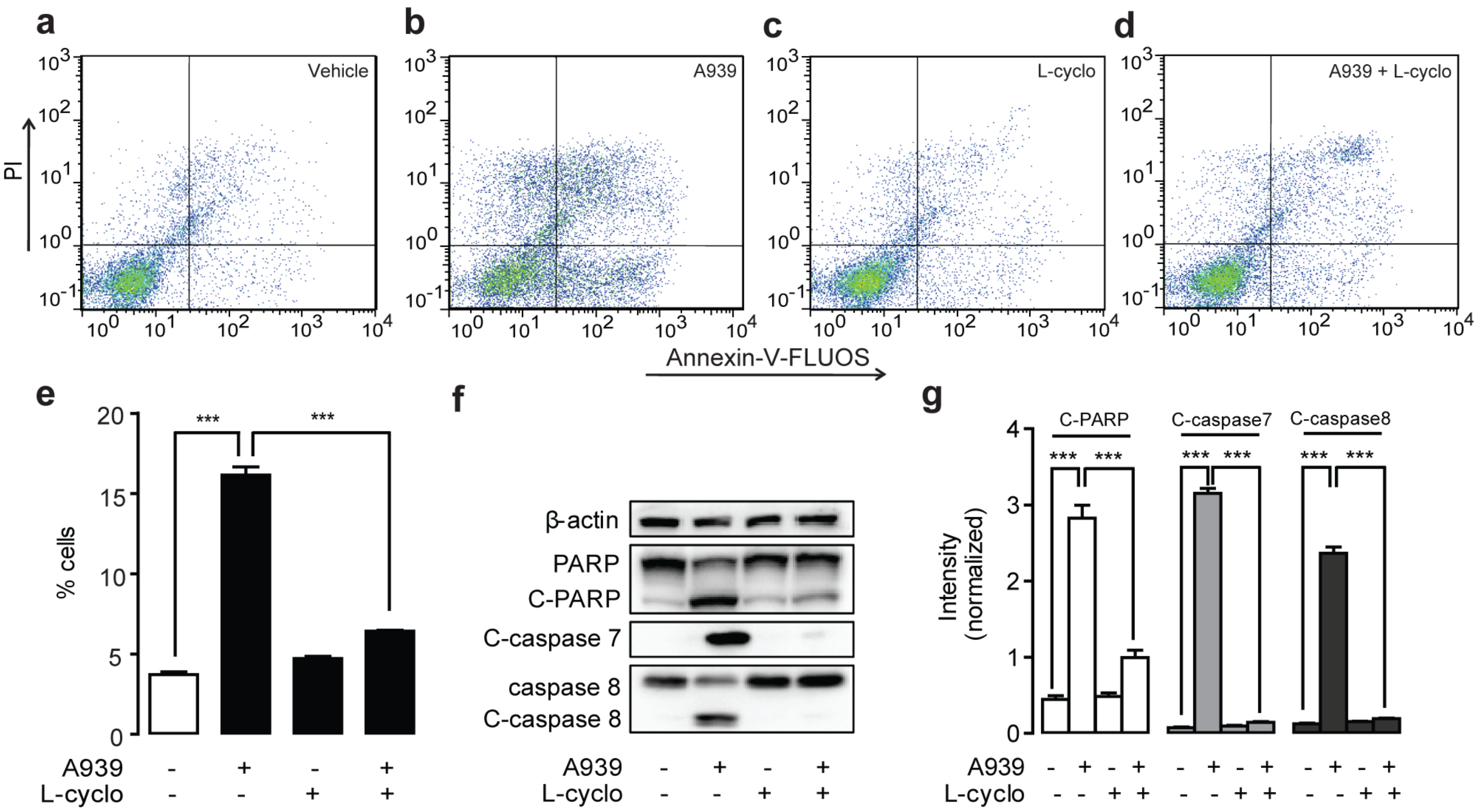

Fig. S9. L-cyclo reversed A939-induced cell apoptosis in human colorectal cancer Colo205 cells. (**a-d**) The effect of vehicle (**a**), 0.2 μM A939 (**b**), 1 mM L-cyclo (**c**), and 0.2 μM A939 combined with 1 mM L-cyclo (**d**) on cell apoptosis, determined with Annexin-V-FLUOS/PI by FACS. (**e**) Quantitative analysis of cell apoptosis from **a-d** data. (**f, g**) The effect of 0.2 μM A939 without/with 1 mM L-cyclo on the protein expression levels of PARP, Cleaved-PARP, Cleaved-caspase 7, caspase 8 and Cleaved-caspase 8, accessed by western-blot (**f**) and quantification of protein expression levels normalized with β-actin (**g**). $^{***}P < 0.001$, One-way ANOVA analysis, $n = 3$.

Supplementary Figure 10

Chen et al.

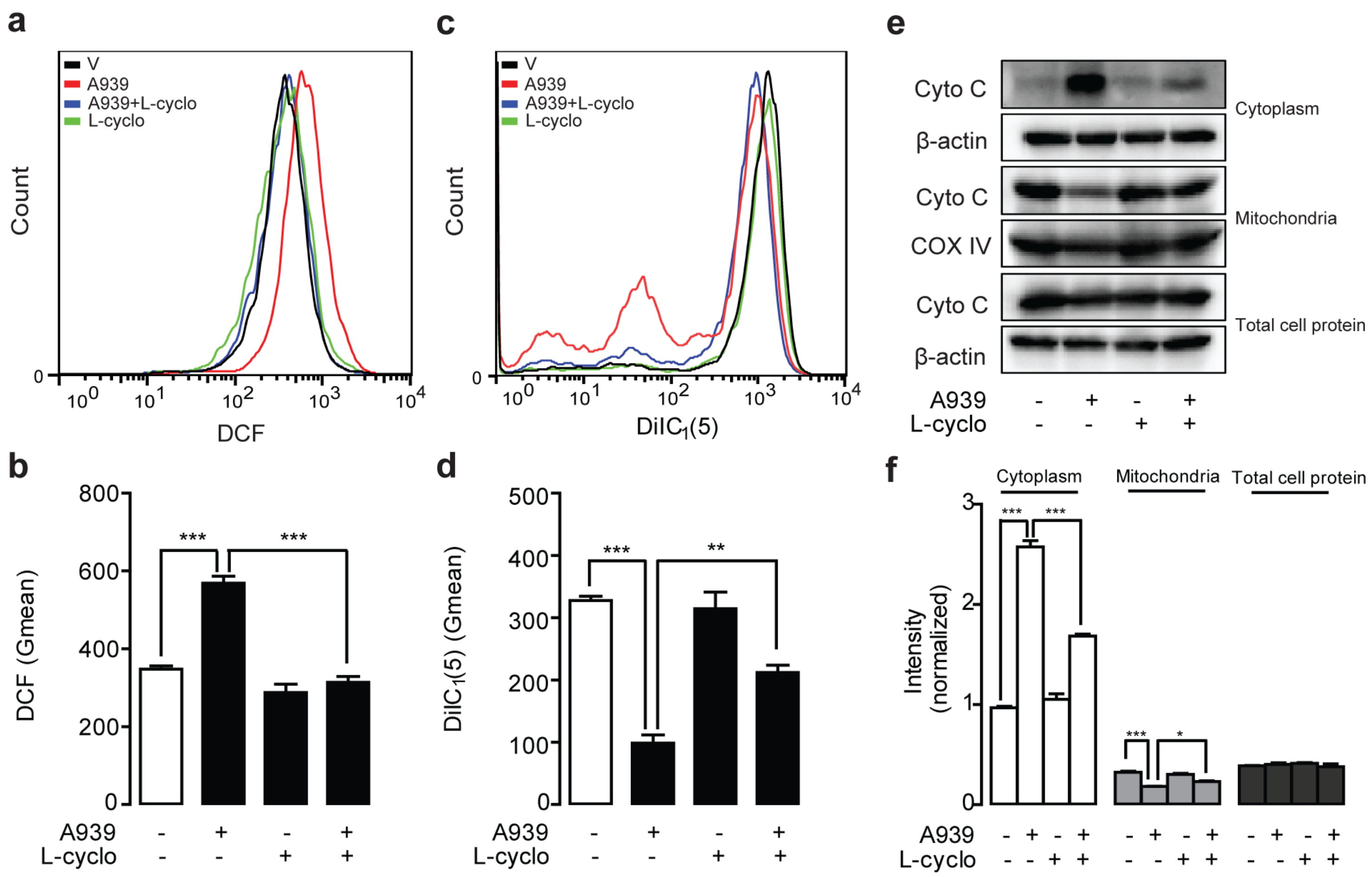

Fig. S10. L-cyclo alleviated A939-induced mitochondria dysfunction in Colo205 cells. The effect of 0.2 μM A939 without/with 1 mM L-cyclo on (**a, b**) cellular ROS production, detected with DCF fluorescence in flow cytometer (**a**) and quantification of ROS release with geometrical mean values (Gmean) (**b**); (**c**, **d**) mitochondrial membrane potential, detected with $DiIC_1(5)$ dye with flow cytometer (**c**) and density quantification (**d**); and (**e**, **f**) cytochrome C protein contents in cytoplasm fraction, mitochondrial fraction, and entire cell, detected by western-blot (**e**) and quantitative analysis by normalizing with *β*–actin or COX IV (**f**). $^{*}P < 0.05$, $^{**}P < 0.01$, $^{***}P < 0.001$ *vs* vehicle treatment, One-way ANOVA analysis, $n = 3$.

Supplementary Figure 11

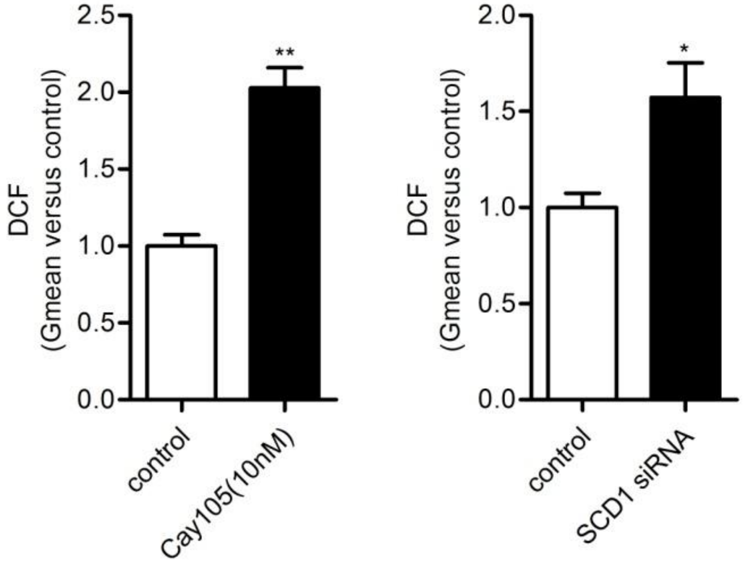

Fig. S11. Cay105 and SCD1 siRNA induced high production of ROS in LOVO cells. LOVO cells were respectively treated with Cay105 (10 nM) or SCD1 siRNA (10 nM), and cellular ROS production was detected with DCF fluorescence in flow cytometer, which was quantified with geometrical mean values (Gmean). $^{*}P < 0.05$, $^{**}P < 0.01$ *vs* control group, Student's *t*-test,, $n = 3$.

Supplementary Figure 12

Chen et al.

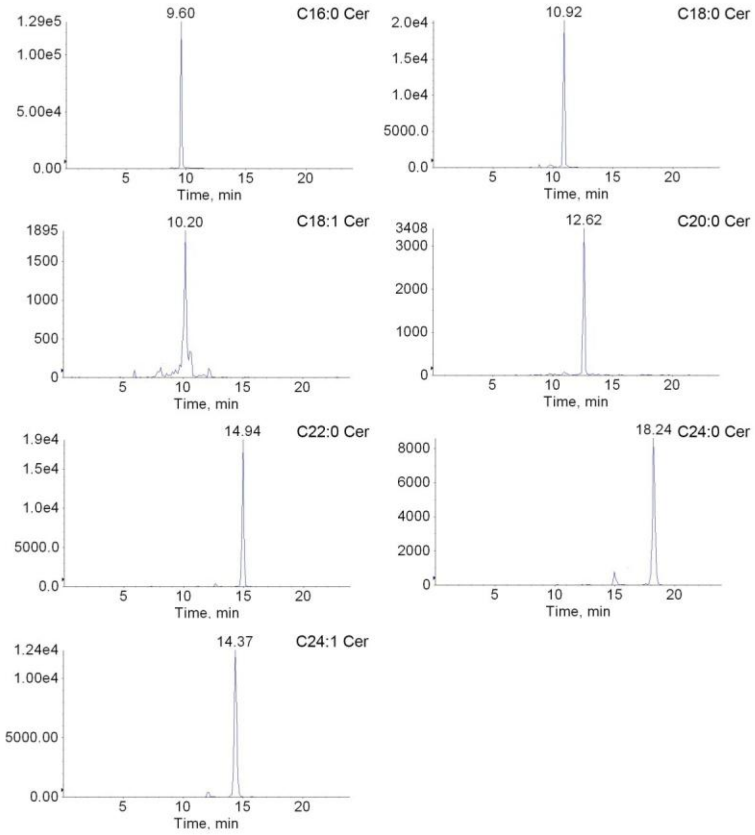

Fig. S12. LC-MS/MS diagrams of ceramides.
